# Personal responsibility for health? A phenomenographic analysis of general practitioners’ conceptions

**DOI:** 10.1080/02813432.2021.1935048

**Published:** 2021-06-15

**Authors:** Joar Björk, Terese Stenfors, Niklas Juth, A. Birgitta Gunnarsson

**Affiliations:** a Stockholm Centre for Healthcare Ethics (CHE), LIME, Karolinska Institutet, Stockholm, Sweden; b Department of Research and Development, Region Kronoberg, Växjö, Sweden; c Department of Learning, Informatics, Management and Ethics (LIME), Karolinska Institutet, Stockholm, Sweden; d Institute of Neuroscience and Physiology, Section for Health and Rehabilitation, The Sahlgrenska Academy, University of Gothenburg, Gothenburg, Sweden

**Keywords:** Attitudes of health personnel, responsibility, general practitioners, qualitative research, phenomenography, health priorities

## Abstract

**Objective:**

To analyse and describe general practitioners’ perceptions of the notion of a ‘personal responsibility for health’.

**Design:**

Interview study, phenomenographic analysis.

**Setting:**

Swedish primary health care.

**Subjects:**

General Practitioners (GPs).

**Main outcome measures:**

Using the phenomenographic method, the different views of the phenomenon (here: personal responsibility for health) were presented in an outcome space to illustrate the range of perceptions.

**Results:**

The participants found the notion of personal responsibility for health relevant to their practice. There was a wide range of perceptions regarding the *origins* of this responsibility, which was seen as coming from within yourself; from your relationships to specific others; and/or from your relationship with the generalized other. Furthermore, the *expressions* of this responsibility were perceived as including owning your health problem; not offloading all responsibility onto the GP; taking active measures to keep and improve health; and/or accepting help in health. The GP was described as playing a key role in shaping and defining the patient’s responsibility for his/her health. Some aspects of personal responsibility for health roused strong emotions in the participants, especially situations where the patient was seen as offloading all responsibility onto the GP.

**Conclusion:**

The notion of personal responsibility for health is relevant to GPs. However, it is open to a broad range of interpretations and modulated by the patient-physician interaction. This may make it unsuitable for usage in health care priority settings. More research is mandated to further investigate how physicians work with patient responsibility, and how this affects the patient-physician relationship and the physician’s own well-being.

**Key Points**
- The notion of personal responsibility for health has relevance for discussions about priority setting and person-centred care.
- This study, using a phenomenographic approach, investigated the views of Swedish GPs about the notion of personal responsibility for health.
- The participants found the notion relevant to their practice. They expressed a broad range of views of what a personal responsibility for health entails and how it arises. The GP was described as playing a key role in shaping and defining the patient’s responsibilities for his/her health.
- The notion was emotionally charged to the participants, and when patients were seen as offloading all responsibility onto the GP this gave rise to frustration.

## Introduction

Fair priority setting is vital to achieving justice and efficiency in health care [1]. In Sweden, three hierarchically ordered normative principles make up the so-called ‘ethical platform for priority setting’: the principle of human dignity, the principle of needs and solidarity, and the principle of cost-efficiency [2]. These principles should inform priority setting decisions at all levels of health care, including primary health care [2]. Much of the literature on priority setting in health care has focused on the hospital setting, but general practitioners (GPs) also play an important role in priority setting [3].

A contested issue in the debate on priority setting is whether a patient’s priority for treatment should reflect his/her degree of responsibility for the disease [4]. In plain language: should the fact that a patient is, for instance, a long-time smoker affect his/her access to treatment for a disease where smoking is a known risk factor, compared to a patient with the same disease who never smoked? The human dignity principle, included in the Swedish ethical platform for priority setting, expressly prohibits taking previous behaviour into consideration when setting priorities. (Expected *future* behaviour may be taken into account if it will strongly affect the outcome of interventions) [2]. However, The National Centre for Priority Setting in Health Care in 2007 suggested that the platform be augmented with a ‘principle of responsibility’. According to this suggested principle, ‘patients whose imprudent health behaviour has contributed to the establishment of their disease (should) be down prioritized for treatment in comparison with patients with no such history of imprudence in health’ [5]. The suggestion was criticized, however, and the Swedish ethical platform continues to reject such responsibilization of patients [6].

Empirical research of people’s attitudes on this topic provides a mixed picture. When asked outright, most members of the Swedish general population, as well as Swedish physicians, reject accounting for responsibility in priority setting decisions [7]. However, one previous study of Swedish physicians showed that respondents were more willing to offer lung cancer treatment to a non-smoking patient with lung cancer than to a smoking patient with the same disease [4]. Many previous studies show that health care personnel find that patients ought to take responsibility for their health, yet do not explain what is meant by taking responsibility [8–11]. A recent Norwegian vignette study of hospital specialists showed support for a principle of health responsibility, yet not for letting such a principle inform priority setting [12]. There is, thus, a certain confusion as to what Scandinavian physicians think about letting responsibility for health inform priority setting decisions, and very little is known about how they conceptualize the notion of personal responsibility for health.

The notion of responsibility for health is of particular interest to GPs. Within family medicine, there is special emphasis on establishing and maintaining a strong personal relationship with the patient [13,14]. Therefore, GPs may be well placed to judge whether a patient has behaved responsibly health-wise. At the same time, GPs passing such judgment may risk hurting their therapeutic allegiance to the patient. Furthermore, in their role as gatekeepers to secondary care, GPs would be of strategic importance if the notion of responsibility for health were to be implemented in priority setting policy.

The aim of this study was therefore to reach an in-depth understanding of GPs’ conceptions of what it may mean to speak of a ‘personal responsibility for health’.

## Material and methods

### Design

This study had a phenomenographic approach and used individual interviews as a method of data collection. Phenomenography is a research approach used to explore participants’ conceptions regarding a particular phenomenon, the collective variations of conceptions in the studied group, and the interrelations between such variations [15].

### Participants and recruitment

In Sweden, GPs are specialized in general medicine and work at primary care centres which may be operated by the county council or by a private employer. In either case, primary care is funded by tax revenue. Participants for this study were drawn from the body of GPs in a county in the south of Sweden.

The potential participants were chosen by purposeful sampling in order to include a breadth of conceptions regarding the phenomenon, in line with phenomenographic tradition [16]. The sampling was performed by the Regional Department of Competence in Family Medicine and Primary Health Care, and a variety was sought regarding age, gender, urban/rural area of service and employment by public/private employer.

The potential participants were approached by e-mail. In the first round, 15 e-mails were sent and 11 responded, accepting to participate in the study. The remaining four did not answer despite one e-mail reminder. The non-participants did not differ systematically from the participants in any of the above factors.

After the first 11 interviews had been made, it was not obvious whether a satisfying level of thematic saturation had been reached. Therefore, the participants were contacted again and asked to provide names of further possible participants who might represent outlier views. In round two, three more potential participants were contacted, again by e-mail. The snowball process used to identify these possible participants was made clear to them. All three consented to participate. After these three participants had been interviewed, no new conceptions were identified. Hence it was concluded that sufficient data had been gathered. The final number of participants was 14. Participant characteristics are presented in Table 1.

**Table 1.** Participant characteristics (*n* = 14).

Sex	
Male/female	6/8
Age (years)	
Mean	49
Min-max (range)	35–71
Area of service	
Urban/rural	7/7
Employer	
Public/private	11/3

### Data collection

The interviews were conducted face-to-face at a venue chosen by the participant, most often their office. All interviews were conducted by the first author (JB). The interviews were conducted between December 2019 and May 2020. The interviews were audio-recorded and transcribed verbatim. The interviews lasted between 23 and 49 min. There were no repeat interviews.

The interviews were semi-structured, based on an interview guide focusing on ways in which lifestyle may influence health; the participants’ attitudes towards unhealthy lifestyles; whether the participants think it is reasonable to claim that there is a responsibility for health and if so what that entails; and, finally, whether such responsibility should affect priority setting in health care. No definition of ‘personal responsibility for health’ was given by the interviewer, instead, participants were encouraged to develop their own notions of the concept.

True to the nature of phenomenography, interview questions were kept open so that the participants could expand on any issues they found most relevant. Reformulated questions were used to improve the understanding of participants’ points of view. Ideals for interviews set down by Ashworth and Lucas were adhered to [17]. In particular, follow-up questions were used to encourage further articulation of the participants’ thoughts [18].

### Data analysis

The aim of data analysis was to inductively categorize qualitatively different conceptions of the phenomenon: ‘personal responsibility for health’. The data analysis was undertaken in accordance with the seven-step protocol for phenomenographical analysis recommended by Dahlgren and Fallsberg [19] (see Table 2), with the aim of producing an outcome space representative of the data as a totality.

**Table 2.** Seven-step protocol for phenomenographic analysis after Dahlgren and Fallsberg [19].

Familiarisation *The transcripts are carefully read and reread* Condensation *The most significant statements are selected to give a short but representative version of the entire dialogue concerning a certain phenomenon* Comparison *The selected excerpts are compared in order to find sources of variation or agreement* Grouping *Answers which appear to be similar are put together* Articulating *A preliminary attempt is made to describe the essence of the similarity within each group of answers* Labelling *The various categories are denoted by constructing a suitable linguistic expression* Contrasting *The obtained categories are compared with regard to similarities and differences*

The main analysis was conducted by the first author JB. After condensation (step 2), TS, NJ and ABG individually read parts of the transcribed text to assess whether JB’s suggested condensation captured the essential aspects of the material. After JB had worked through steps 1–5 and presented preliminary categories, the other authors scrutinized the preliminary categories, working through steps 3–5 individually. At steps 2–4, special care was taken to capture also what each particular statement implied in terms of underlying assumptions [20]. Through a consensus procedure, the preliminary categories were reworked. Trustworthiness was aimed at by using previously established protocols (see above). Furthermore, all authors continually discussed their presuppositions and how these may have influenced the interpretation of the data [21].

### Ethics

All participants were given written and verbal information about the study and gave their written informed consent to participate. Participation was voluntary and could be terminated at any time and confidentiality was assured. The study design including the interview guide was approved by the Ethical Review Board, Sweden (DNR 2019-02275).

## Results

All participants expressed views that presuppose the existence or relevance of some kind of personal responsibility for health. The outcome space was constructed with two qualitatively separate parts, illustrating discrete aspects of the understanding of such responsibility.

**Figure 1.**
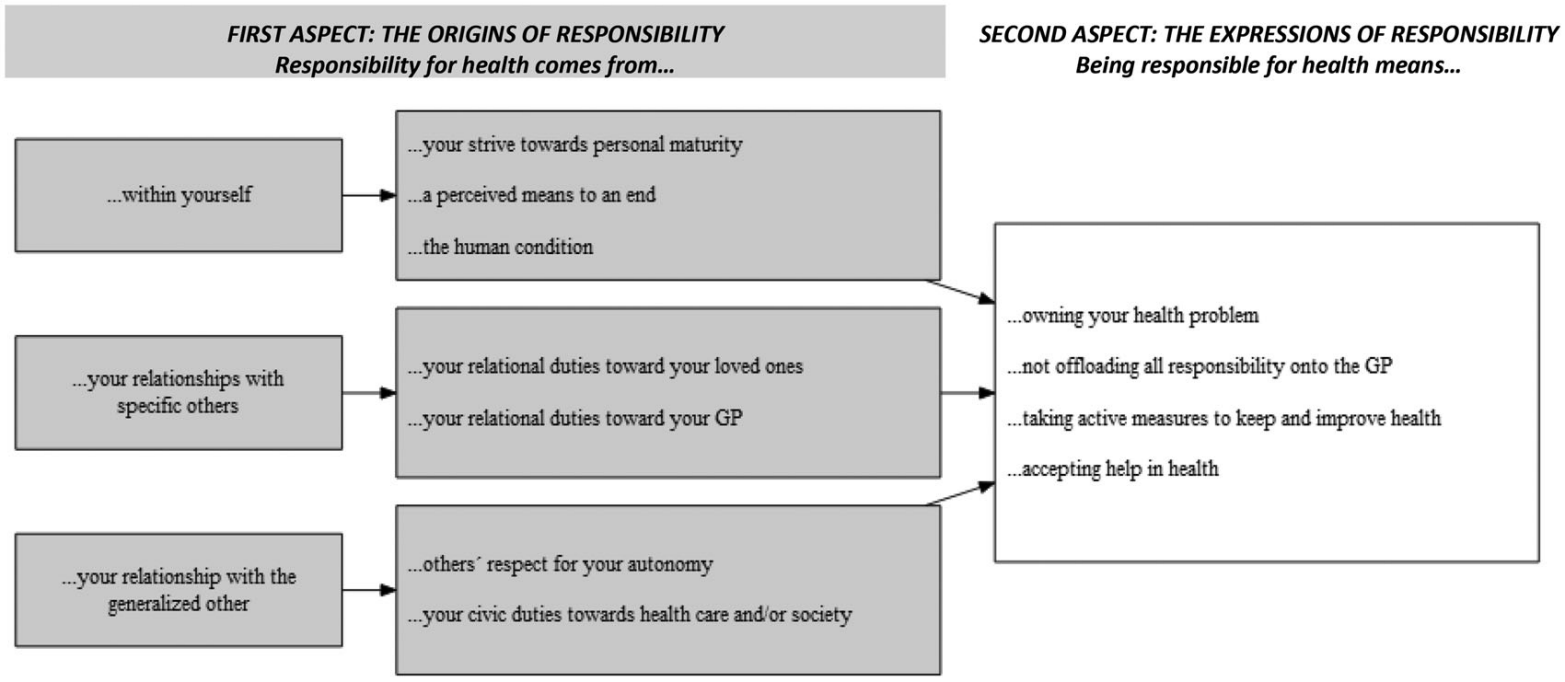
Outcome space – participants’ understanding of a ‘personal responsibility for health’.

The first aspect in the outcome space concerns *the origins of responsibility*, which include the interrelated issues of why it is that people have responsibility for their health, and towards whom this responsibility is directed. These issues are presented together as several conceptions saw responsibility as arising out of the demands of specific relationships (to yourself or to others). The second aspect concerns *the expressions of responsibility* and deals with what it means to have such responsibility (see Figure 1). Each aspect contains several categories of description. There are no obvious conceptual links between the categories in the first aspect and the second aspect. Instead, categories from the first aspects and second aspects can be freely combined.

Below, the different conceptions of the phenomenon will be described using a standardized literary form, where content is expressed in terms of ‘your’ responsibility for ‘your’ health. This mirrors a common way of expression among the participants. Some participants used ‘you’ as a way of mimicking a conversation with a patient, whereas others used ‘you’ in the general sense (as in ‘somebody’ or ‘one’). Hence, the word ‘you’ should be read to mean either a patient or a person in general. Furthermore, no participant ascribed different responsibilities to patients versus non-patients, so all conceptions of responsibility for health should be understood as being applicable to both patients and people in general. The participants alternated between talking specifically about responsibility for *health* and, more generally, for *welfare*. When this was probed by follow-up questions, no participant expressed differentiated views on this topic. The implication was rather that your responsibility for your health is included in your general responsibilities for your welfare. Many participants spoke also of other relevant parties’ responsibility for your health (peers, society, commercial actors, etc.). In the following, such conceptions will not be further explored as the focus here is on your responsibility for your health. Finally, although possible differences of nuance exist between ‘having responsibility’ and ‘taking responsibility’, no participant differentiated between these expressions. In keeping with phenomenographic tradition, all substantive, qualitatively different conceptions of responsibility for health will thus be explicitly pointed out below, rather than by using a complex and differentiated nomenclature alien to the participants [19].

### First aspect: the origins of responsibility

#### Responsibility for health comes from within yourself – as part of your strive towards personal maturity

Taking responsibility for your health is perceived as being a mature person:

I guess it’s a sign of maturity to take responsibility for yourself and your well-being… (GP 1)

It is made clear that it is in a sense optional to strive for maturity, yet the conception has a normative implication to the effect that you *ought* to strive for maturity. This, in turn, is seen as part of a larger striving to become the best possible version of yourself:

At the end of the day, everybody is responsible to take care of themselves and their capacities, and not waste their opportunities – that’s a responsibility, or challenge, that every living human has. (GP 8)

#### Responsibility for health comes from within yourself – as part of the human condition

The conceptions in this category again perceive your responsibility for your health as essentially a private matter, but the attitude here is purely descriptive. Here responsibility (including responsibility for your health) is seen as a by-product of the fact that, as humans, we make choices. At the same time, it is recognized that outside factors may affect your capacity for free action:

Being human is to take responsibility, that is part of the provisions. We have responsibility for ourselves and for those around us, I think, to various degrees depending upon your capacities. (GP 6)

#### Responsibility for health comes from within yourself – as a perceived means to an end

Here taking responsibility for your health, or merely assuming that you have a responsibility for your health, is done in order to attain other goals. The phenomenon is thus seen as a socio-psychological construction playing an instrumental role to the individual’s benefit. The phenomenon may function this way without your awareness of it, or you may be fully aware that a leap of faith is at play:

Your life will be more meaningful if you choose to believe that you have your own will and a possibility to affect your life. (GP 5)

#### Responsibility for health comes from your relationships with specific others – as part of relational duties to your loved ones

This and the following conceptions see your responsibility not as coming from within, but from your relationships with others. Here, your responsibility for your health arises as a consequence of your bonds to those close to you. Conversely, then, there is no responsibility for health in the absence of close relationships:

If you are quite isolated then I think it (=responsibility) doesn’t apply, but as soon as another enters the picture/…/then there’s a relationship and then things change. Then you have a responsibility to/…/make sure you last a while longer. (GP 13)

#### Responsibility for health comes from your relationships with specific others – as part of relational duties to your GP

Here your responsibility for your health is seen as arising (at least in part) from your relationship with your GP. More to the point, whenever you and your GP reach a treatment agreement, you become responsible for (that facet of) your health.

Yes, I’ll help you, but on the condition that you act this way or that… (GP 14)

However, it should be noted that all participants who expressed this conception also stated that your responsibility towards your GP is of a lesser magnitude than other responsibilities, such as towards yourself or others (other than the GP).

#### Responsibility for health comes from your relationships with the generalized other – as part of their respect for your autonomy

By ‘the generalized other’, it is intended both people to whom you have no personal relationships and your impression of these persons’ expectations of you. This category relies upon an implicit link between being held responsible for your actions on the one hand and being treated as a free, autonomous individual on the other. The link between responsibility and freedom provides an obligation for others to treat you as responsible for your actions:

Everybody owns their own life and you take responsibility for your own life… if I were to step in and take responsibility (for somebody else’s life) then in a way I belittle that individual’s own abilities. (GP 4)

The origin of your responsibility, here, is wholly external as it comes from others’ duty to treat you as one who is responsible.

#### Responsibility for health comes from your relationships with the generalized other – as part of your civic duties

Again, your responsibility is seen as arising from your relationship with the generalized other but here the notion of responsibility for health is subsumed under the larger notion of civic duties:

You have a responsibility to do what you can to be something that contributes to society, rather than just letting yourself go to waste. (GP 13)

There was variation regarding the precise nature of such civic duties, with some participants focussing on general prudence and others or the risk of incurring opportunity cost for others:

I simply think you shouldn’t cheat the system. (GP 7)

Of course, if I don’t take care of myself and if I am often ill, it will be expensive to society. It will be expensive to healthcare, it will be expensive to the taxpayer. It will be expensive not only in money, but it will also set a precedent (for others). (GP 8)

As with your responsibility towards your GP, your responsibility to society was expressly mentioned as being of lesser magnitude than other responsibilities.

### Second aspect: the expressions of responsibility

While the first aspect concerns views on *why* you have personal responsibility for your health, the second aspect deals with *what it means* to have such responsibility. It should be pointed out that whereas the participants adhered to one or at most a few of the views presented in the first aspect, many participants expressed several of the views presented in the second aspect. Thus, although the notions below are qualitatively separate, they are not mutually exclusive but should be seen as forming a collective whole (even to the individual participant).

#### Being responsible for health means owning your health problem

Being responsible for your own health is seen as realizing that your health is primarily in your own hands. This entails, further, the realization that fixing the problem may come at a cost to yourself:

It is the individual’s responsibility that if this person wishes to improve their situation or how they feel with their pain issue, then this personal commitment is necessary. (GP 9)

Importantly, this view enables you to opt-out of optimizing health. As long as you own up to your choices, you may still be perceived as taking responsibility:

A patient with diabetes who isn’t ready to change his/her behaviour but who says’ I’ll take this risk’, well I think that patient has also taken responsibility. (GP 7)

#### Being responsible for health means not offloading all responsibility onto the GP

This category contains notions that are closely related to those in the previous category. Here, however, the opposite of taking responsibility for your health is not seen as a rejection of responsibility in general, but rather as the specific act of offloading your responsibility onto the GP. This, therefore, is a relational notion, centred on the interplay between patient and GP. Implied is a division of labour when it comes to responsibility so that the patient does one part and the GP another:

But some like to put the responsibility on us, like’ you’re my doctor, you should fix this’/…/then you must say ‘of course I can treat this/…/but the underlying problem, that ball is in your court’. (GP 8)

#### Being responsible for health means taking active measures to keep and improve health

Here, taking responsibility is seen as a matter of taking action health-wise. Relevant actions include monitoring your health and your lifestyle, as well as improving unhealthy behaviour. However, being responsible does not necessitate perfect behaviour so much as making an honest effort to stay healthy:

When it comes to personal responsibility for health, I think that means that you do what you can, to maintain your health. (GP 14)

#### Being responsible for health means accepting help in health

The underlying assumption here is that you need help to stay healthy, and taking responsibility for your health means seeking and utilizing such help. Conversely, it is seen as a failure of responsibility if you do not comply with the help offered:

If the patient does not follow my instructions, then I think that is the patient’s responsibility. (GP 13)

This category of conceptions is not purely descriptive but contains strong emotional matter. The participants expressed how your failure to behave responsibly, in this sense, may affect the GP strongly:

And then you feel that the patients are not being responsible when they don’t pay attention to it (=the GP’s advice) and you perceive that as rather spiteful. (GP 14)

## Discussion

This study indicates that GPs find it appropriate to speak of personal responsibility for health and that such responsibility has relevance for health care. However, as witnessed by the breadth of the outcome space, there was a wide variety of conceptions regarding the origins as well as the expressions of responsibility for health. The richness of conceptions was evidenced not only in the group total but also among individual participants, showing that the notion of responsibility for health is highly complex.

Responsibility is variously seen as something which you owe yourself, to your loved ones, and/or to health care/society. Responsibility may also arise from the physician-patient relationship, as an agreement or kind of contract. Indeed, many of the conceptions expressed imply some sort of ‘responsibility negotiation’, implicit or explicit, taking part between GP and patient. Being responsible for your health may further mean that you own your health problem, take active measures to keep and improve your health, accept help in health and/or that you do not offload all responsibility onto your GP.

The phenomenon under scrutiny was ‘personal responsibility for health’, and we had expected that the GPs would speak mainly about their patients’ responsibilities. This they did, but also about themselves (or other physicians). Thus, it appears the GP him-/herself is of great importance to the topic of responsibility, for instance as a conversation partner in the construction and definition of a patient’s responsibilities. This sets this study apart from some of the more abstract philosophical contributions to the literature on this topic [22–25]. Far from being abstract or formal, responsibility is here seen as a richly contextualised and relational concept. Responsibility is partly seen as an artefact of the doctor-patient relationship, but it also has a profound influence on the same relationship.

In addition to its conceptual richness, the topic was emotionally engaging to the participants. The notion of responsibility for health evoked not only descriptive but also strongly normative connotations. When, for instance, the participants described patients who were seen as offloading their responsibility onto their GP, they were clearly upset. This, then, marks another aspect in which the notion of responsibility involves not only the patient but also the GP.

While we know of no previous studies on this precise topic, the results tie into several ongoing debates. The involvement of the GP him-/herself in creating or articulating responsibility may be analysed against a backdrop of current discussions about empowerment, shared decision making and patient-centred care. Often these discussions include an increase in patients’ responsibility for his/her health as a sub-goal of modern medicine [26,27]. At the same time, there may be a goal conflict at play here, as a strong agenda on the physician’s part (for instance: an agenda to increase the patient’s sense of responsibility) may in itself hinder truly shared decision making [28–30]. It may thus be relevant to ask: Who sets the standard for responsibility, the patient or the physician? As pointed out in the beginning, there are also obvious tensions between the ‘responsibilization’ of patients and issues of distributive justice [31]. It is partly for this reason that the Swedish priority-setting platform explicitly forbids some implications of personal responsibility for health [2]. A simple take-home message may be: Tread softly, GP, on the field of responsibility.

Another reason to tread softly is that general medicine is committed to a holistic view of the patient, building on the bio-psycho-social model [32,33]. The rich data on social and epigenetic determinants of health and health behaviour would indicate that a person’s own responsibility for health can never be more than part of a larger story [34,35]. Some but not all conceptions in this study reflect this. It may be argued that failure to account for relevant limitations in a patient’s capacity to take responsibility for her health clashes with the idea of seeing the patient holistically. It may further be of hindrance in building a therapeutic alliance with the patient (see more below).

More specifically, some conceptions in this study see taking responsibility for health as adhering to the physicians’ advice. This view is problematic in several ways. Empirical data show that patient adherence to medical advice and medication is low [36], and that physicians are poor at assessing patients’ adherence [37,38]. Unfortunately, patient education and patient self-management programs designed to improve adherence show at best modest benefits [36,39]. In the light of this, a salient question is whether (some) physicians are capable of assessing responsibility? Furthermore, to the extent that physicians negotiate responsibility with the hope of increasing adherence, this may not be a very effective measure. Finally, as so many patients obviously fail to adhere to advice and medication, responsibility-as-adherence may be an unnecessarily elitist notion. Instead, it may be wiser, ethically and pragmatically, to see adherence (and perhaps also responsibility for health) as part of a shared undertaking rather than a feature of the individual patient [40,41].

Meanwhile, the emotional involvement triggered in the GPs by responsibility issues raises other questions. For instance: What is the ideal level of GPs’ emotional involvement? Rudebeck writes of a delicate ‘balance between involvement and detachment’, and interestingly claims that ‘detachment is necessary to take responsibility’ (discussing the physician’s own responsibility) [14]. The balance between involvement and detachment may be of importance not only to the quality of health care but also to the well-being of the physician him/herself, as has been argued in recent writing on physicians’ clinical empathy and burn-out [42,43]. Further studies are needed to elucidate the possible interrelations between responsibility issues in the clinic, physician well-being and clinical empathy.

The conceptions in this study, taken as a whole, provide a more tolerant view of the less-than-responsible patient than some previous survey studies (see for instance [44]). No participant in this study expressed support for the down-prioritization of ‘irresponsible’ patients. One reason may be that immediate, intuitive opinions on responsibility for health, typically elicited by the survey format, differ from more deliberated views typically elicited by formats such as used in this study, which encourage deliberation and reflection [45].

The very heterogeneous nature of GPs understanding of responsibility for health may make the notion unsuitable for use in policy-making. If GPs are to implement a responsibility-based policy, they will first need to unlearn their currently differing views of what responsibility means, and then adopt a common understanding. This may prove a difficult challenge.

### Methodological reflections

Among the strengths of this study are the novel content and the usage of the phenomenographic method, which is especially suited to capturing participants’ thoughts about abstract concepts and ideas [46].

Credibility was aimed at by using investigator triangulation, where each co-author contributed with differing pre-suppositions and differing expertise, Region Kronoberg is a physician and clinical ethicist with thorough experience of structured ethical reflections with health care staff. TS is a Medical Education researcher with expertise within qualitative research methodology, including phenomenography, and in the lifelong learning of health care professionals. NJ is a bioethicist with expertise in conceptual and normative analysis as well as descriptive content analysis. ABG is an occupational therapist and associate professor in medical sciences, with expertise in qualitative research methodology including phenomenography and research in primary health care settings.

One worry was that any way of asking the participants about their views of patient’s responsibilities for their health would insinuate that it makes sense to speak of such a responsibility. The practical solution was to ask: ‘Some claim that it makes sense to speak of a personal responsibility for health, what do you make of that?’. As all conceptions in this study strongly indicate that the notion of patients’ responsibility for their health is a meaningful concept, it seems unlikely that this was an artefact of the way the question was asked.

Dependability was increased by the usage of a well-described analysis protocol [19].

The transferability of this study was increased by the usage of a purposive sampling strategy undertaken by an external party. The additional snowball sampling, undertaken to recruit participants who might represent outlier views, presumably increased variety in responses. The results are likely transferable to Swedish GPs in other regions, but less likely to be transferable to other medical specialties due to the particular nature of GPs work. However, the results have relevance for broader discussions regarding for instance consultation models and priority setting.

### Implications for further research

As conceptions of responsibility towards oneself, one’s family and one’s society are presumably culturally dependent, it would be interesting to repeat this study in another country, preferably with a different health care system. Other contrasting points of departure would be to investigate how physicians from other medical specialties, or patients, think about responsibility for health.

Ways to broaden understanding in this area would be to investigate how GPs conceive of the various modulators of patients’ capacities to be responsible – and how GPs may work with these modulators. On a related note, it may be investigated what help GPs feel they need to work with patients who are perceived as irresponsible, and the frustration that this may engender. This issue is essential so that GPs can provide efficient and compassionate care to patients across the scale of (perceived) responsibility.

A further idea would be to use participatory study methods to describe the ‘responsibility negotiation’ between physician and patient implied by this study.

## Conclusion

In this analysis of interview data with Swedish GPs, all conceptions view the notion of a patient’s responsibility for his/her health as relevant. The conceptions differ as to the *origins* of this responsibility, which are seen as lying within the patient him-/herself, within the relationship to loved ones or the GP, or as part of civic duties. The conceptions also differ as to the *expressions* of responsibility, which range from attitudes of ‘owning the problem’ to actions of accepting help in health. The participants offered not only descriptive information about responsibility but also normative. For instance, when patients were seen as avoiding responsibility by offloading all responsibility onto the GP, this gave rise to frustration in the GPs. The conceptions presented emphasize the role played by the GP and the physician-patient relationship in shaping and defining the patient’s responsibilities for his/her health.
